# Bilinguals Produce Pitch Range Differently in Their Two Languages to Convey Social Meaning

**DOI:** 10.1177/00238309221105210

**Published:** 2022-07-15

**Authors:** Elisa Passoni, Esther de Leeuw, Erez Levon

**Affiliations:** Department of Linguistics, Queen Mary University of London, UK; Center for the Study of Language and Society, University of Bern, Switzerland

**Keywords:** Pitch range, bilingualism, sex, individual gender identity, politeness

## Abstract

We investigated whether expression of social meaning operationalized as individual gender identitity and politeness moderated pitch range in the two languages of female and male Japanese-English sequential bilinguals. The bilinguals were resident in either London (UK) or Tokyo (Japan) and read sentences to imagined addressees who varied in formality and sex. Results indicated significant differences in the pitch range of the two languages of the bilinguals, and this was confirmed for female *and* male bilinguals in London *and* Tokyo, with the language differences being more extreme in the London bilinguals than in the Tokyo bilinguals. Interestingly, self-attribution of masculine gender traits patterned with within-language variation in the English pitch level of the female bilinguals, whereas self-attribution of feminine gender traits patterned with within-language variation in the English pitch level of the male bilinguals. In addition, female *and* male bilinguals significantly varied their pitch range in Japanese, but not in English, as a function of the imagined addressees. Findings confirmed that bilinguals produce pitch range differently in their languages and suggest that expression of social meaning may affect pitch range of the two languages of female and male bilinguals differently.

## 1 Introduction

Sociolinguistic research has shown that phonetic variation has social meaning (Eckert & Labov, 2017) and that monolinguals vary their pitch range^1^ as a way of drawing on these meanings to make social moves and construct identities (Levon, 2018; Lewis, 2002; Podesva, 2007). Moreover, research has indicated that speakers of different languages use characteristically different pitch ranges (Keating & Kuo, 2012; Mennen et al., 2012) because of linguistic and sociocultural constraints (Dolson, 1994; Yuasa, 2008). Furthermore, within-speaker language-specific pitch range variation has been attested in bilinguals (Altenberg & Ferrand, 2006; de Leeuw, 2019; Loveday, 1981; Ohara, 1992; Ordin & Mennen, 2017); however, less is known about how bilinguals vary their pitch range to (dis)align with the conventionalized social norms of their two languages, if at all. To fill this gap, we investigated sources of variation in the pitch range of the two languages of Japanese-English sequential bilinguals in London (UK) and Tokyo (Japan).

For the purpose of this work, we define social meaning as “the conventional association of distinctions in the world with distinctions in the [phonetic] form” (Eckert & Labov, 2017, p. 3). Due to its conventional nature, social meaning is dependent on a shared cultural common ground (Eckert, 2019), and thus varies across social and language groups. Japanese and British English (English hereafter) are interesting in this regard because of the different meanings the two languages conventionally attribute to high pitch level and wide pitch span (i.e., the two dimensions along which pitch range varies—see section 2.5). In Japanese, a higher pitch level and wider pitch span are considered to index femininity (Hiramoto, 2010; Loveday, 1981; Ohara, 1992, 1999, 2019) and by extension politeness (see Ide, 1982 for the intersection between femininity and politeness in Japanese). In English, the same phonetic features are generally thought to be used to index politeness by *both* females and males (Loveday, 1981). Note that in Japanese and English, politeness involves showing that “one thinks well of others [. . .] and [. . .] does not think too highly of oneself” (Haugh, 2007, p. 88); however, in Japanese, politeness is linked to modesty and being reserved (“politeness-as-deference”; Tsurutani & Shi, 2018, p. 131), whereas in English politeness is linked to friendliness (Pizziconi, 2007).

As described in the literature, Japanese women are stereotypically expected to use Japanese Woman Language (JWL) (Inoue, 2006; Ohara, 2019; Okamoto & Shibamoto Smith, 2007), a “more polite or less vulgar” form of language than men’s speech (Ohara, 2019, p. 238). Prosodically, JWL is implemented by the use of a sustained high-pitched voice (Hiramoto, 2010; Ohara, 1992), on average 40 Hz higher than that of Western women (Van Bezooijen, 1995). Japanese men’s language is traditionally defined in opposition to JWL (Ohara, 2019; Sturtz Sreetharan, 2004). In terms of pitch range, this has been referred to as “a low, almost monotonous, pitch” (Loveday, 1981, p. 83) and a generally “cooler” demeanor (Loveday, 1981; Tsurutani & Shi, 2018). Importantly, there is evidence that (1) Japanese monolingual women and men consider high-pitched voices as more feminine (and more polite) than low-pitched voices (Ofuka et al., 2000; Ohara, 1999) and (2) Japanese monolingual women, but not men, increase their pitch level and widen their pitch span to index politeness (Ohara, 2004).

In English, women have higher pitch than men and, most likely, are expected to be more polite and refined in their speech than men (see Cameron, 2014). However, in English, differences between women’s and men’s speech are perhaps less stark than in Japanese (Ohara, 1999). Importantly, women and men alike have been reported to increase their pitch level and widen their pitch span to communicate friendliness (Loveday, 1981). Therefore, upon acquiring English pitch range norms, Japanese native speakers need to become aware of and navigate the fine distinction in the social meaning each language attributes to high pitch level and wide pitch span.

Previous examinations of pitch variation between the two languages of Japanese-English bilinguals have provided a somewhat inconclusive picture regarding how bilinguals implement these language-specific differences in pitch range. Using a reading task, Loveday (1981) and Ohara (1992) found that *only* female Japanese-English bilinguals^2^ increased their pitch level and widened their pitch span when speaking Japanese compared with English. Graham (2015), in contrast, reported that female *and* male balanced simultaneous bilinguals produced Japanese with a higher pitch level and a wider pitch span than English. A reason for these different results may be the tendency of prior research to focus exclusively on the sex of the participant (i.e., assignment of a biological category at birth; Vincent, 2018) rather than on individual gender identity (i.e., an individual’s (dis)alignment with gender-prototypical norms; Vincent, 2018). Hiramoto (2010) reported that Japanese monolinguals, irrespective of whether they identified as female or male, produced gender-neutral sentence final particles with a higher pitch level and a wider pitch span when instructed to read in “feminine style”. This suggests that Japanese native speakers are aware of the ideological qualities of JWL and can use these pitch realizations to perform femininity, if desired. It is therefore possible that the discrepancies evident across earlier studies of Japanese-English bilinguals result from conflating individuals with various gender identities into overly simplistic sex-based categories. In the present study, we also took individual gender identity into account and examined whether it affects the pitch range of the two languages of female and male Japanese-English bilinguals differently. Furthermore, as outlined in section 2, the bilinguals in London and Tokyo addressed the read sentences to imaginary formal and informal female and male addressees, to assess whether social meaning would be expressed differently through pitch range in different settings.

The research questions we set out to answer were as follows:

*RQ1.* Do female and male Japanese-English bilinguals resident in either London (UK) or Tokyo (Japan) produce pitch range differently in their two languages?
*RQ2.* If so, does expression of social meaning, operationalized as individual gender identity and politeness, influence the pitch range of the two languages of the bilinguals differently?

Results from the previous literature (see references below) led us to develop the following hypotheses. With regard to RQ1, we hypothesized the following:

*H1a*. Japanese pitch level would be higher than English pitch level in females but not males (Ohara, 1992, 1999; but see Graham, 2015);
*H1b*. Japanese pitch span would be wider than English pitch span in females but not males (Ohara, 1992, 1999; but see Graham, 2015).
With regard to RQ2, we hypothesized the following:
*H2a*. There would be no differences in the pitch level elicited by formal-looking and informal-looking addressees in the English of the female and male bilinguals (Ohara, 1999).
*H2b*. There would be no differences in the pitch span elicited by formal-looking and informal-looking addressees in the English of the female and male bilinguals (Ohara, 1999).
*H2c*. Formal-looking addressees would elicit a higher pitch level than informal-looking addressees in the Japanese of the female bilinguals, but not males (Ohara, 1999).
*H2d*. Formal-looking addressees would elicit a wider pitch span than informal-looking addressees in the Japanese of the female bilinguals, but not males (Ohara, 1999).

In addition, it was explored whether (H2e) individual gender identity of the bilinguals, (H2f) sex of the addressee, and (H2g) testing location (London or Tokyo) would explain variation in the two languages of the female and male bilinguals. Through this detailed analysis of pitch range, we were ultimately interested in finding out the extent to which the bilinguals moderated their pitch range to convey social meaning in both of their languages.

## 2 Methods

### 2.1 Participants

Forty-one Japanese-English bilinguals—19 in London (UK) and 22 in Tokyo (Japan) (Table 1)—took part in the study, which was granted ethical approval by both the Queen Mary University of London and Sophia University Research Ethics Committees. Prior to data collection, bilinguals completed an adapted version of the Language Experience and Proficiency Questionnaire (LEAP-Q, Marian et al., 2007); the relevant background information is presented in Table 1.

**Table 1.** Descriptive Statistics of the Relevant Background Information for Participants of Both Testing Locations.

Testing location	Number	Sex of the participant	Biological age	Age of acquisition	L2 proficiency
London (UK)	19	12 females	27 (6.7)	9.9 (3.4)	7.0 (1.4)
		7 males	28 (4.6)	12.2 (1.2)	8.0 (0.9)
Tokyo (Japan)	22	17 females	21 (3.1)	9.1 (3.4)	6.6 (1.8)
		5 males	23 (3.7)	8.2 (3.4)	7.4 (1.9)

*Note*. Means and standard deviations are reported.

Of the 41 bilinguals, 29 were self-reported females and 12 self-reported males. They had all completed or were enrolled in a university course at the time of testing and resided in highly urbanized megacities (see Inoue, 2006, for the effect of residing in an urban center vs. the countryside on Standard Japanese and Ofuka et al., 2000, for dialect exposure in megacities). Participants’ biological age ranged between 18 and 39 years, which ensured minimal biological age-related differences in pitch (see Linville, 1996, for a review).

All participants were sequential bilinguals, who considered Standard Japanese to be their first and dominant language (i.e., the language they felt more confident speaking—L1 hereafter) and English their second and less dominant language (i.e., the language they felt less confident speaking—L2 hereafter). All bilinguals started acquiring English before puberty via formal education (Table 1). They self-reported L2 proficiency on three scales (speaking, understanding, reading) ranging from 0 (*none*) to 10 (*perfect*). A composite proficiency rating was calculated by averaging ratings on the three scales (Schroeder et al., 2015) (Table 1). A series of two-way analysis of variance (ANOVAs) indicated that age of acquisition (AoA) and L2 proficiency did not differ between sex of the participant (female vs. male) and testing locations (London vs. Tokyo) (Appendix A). Independent of whether female or male, bilinguals recruited in London were significantly older than those recruited in Japan, *F*(3, 37) = 13.9, *p* **<** .001, but all were below 40 and this was therefore not considered to impact the results (Linville, 1996). None of the bilinguals reported a history of hearing or speech-language disorders.

### 2.2 Gender identity

We measured bilinguals’ gender identity using the Bem Sex Role Inventory–short (BSRI-short) (Bem, 1979) and the Japanese Gender Role Index (JGRI) (Sugihara & Katsurada, 2002). Both questionnaires are “measures of support for and adherence to cultural gender norms” (Smiler & Epstein, 2010, p. 134), consider femininity and masculinity as sociocultural constructs, and assume that all individuals present both feminine and masculine characteristics to a greater or lesser extent. Given that bilinguals have been reported to activate behavioral expression of personality appropriate to the corresponding linguistic-social context of the language they are speaking (Chen & Bond, 2010) and that we collected speech data in monolingual mode (see section 2.4), we chose to use both questionnaires as they are considered to be tailored to Western Anglophone (BRSI-short) and Japanese (JGRI) gender norms, respectively.

Each questionnaire comprises 30 traits (10 feminine, 10 masculine, and 10 neutral fillers) and respondents are asked to rate the extent to which each item describes themselves on a 7-point scale, ranging from 1 (*never applies*) and 7 (*always applies*). Bilinguals were attributed one masculinity and one femininity score per questionnaire (for a total of four scores) on a continuum from 1 (*low femininity*/*masculinity*) to 7 (*high femininity*/*masculinity*). For both questionnaires, the masculinity score equaled the mean self-rating of all the masculine items and the femininity score the mean self-rating of all the feminine items (Kaźmierski, 2015; see Table 2 for average descriptive statistics and Appendix B for more details). Surprisingly, a series of one-way ANOVAs indicated that sex of the participant (female vs. male) did not predict scores on the femininity and masculinity scales for either of the questionnaires (Appendix B).

**Table 2.** Means and Standard Deviations of the Scores of the Two Gender Identity Questionnaires Divided by Sex of the Participant.

Sex of the participant	*Femininity* BSRI-short	*Masculinity* BSRI-short	*Femininity* JGRI	*Masculinity* JGRI
Female	4.8 (0.8)	4.1 (0.8)	4.5 (1.0)	4.3 (0.9)
Male	4.3 (1.1)	4.5 (1.3)	4.5 (1.0)	3.7 (0.8)

*Note*. BSRI-short: Bem Sex Role Inventory–short; JGRI: Japanese Gender Role Index.

Averages closer to 7 indicate a higher self-identification with femininity and masculinity traits, respectively.

### 2.3 Materials

The dataset consisted of 16 English sentence tokens and their translations in Japanese, taken from Graham (2015). In both English and Japanese, sentences (1) contained a large amount of fully voiced segments, (2) corresponded to a single intonational phrase, and (3) were unmarked in register. To investigate the effect of politeness on the pitch range of Japanese females reported in previous studies (i.e., formal addressees elicited a higher pitch level and a wider pitch span than informal addressees; Ohara, 1999, 2004), we added a visual component to the elicitation procedure in the form of pictures of imagined addressees (for previous work using images to elicit phonetic variation, see Babel, 2009). The images were used as a proxy to elicit the pitch range bilinguals would have used if they encountered those people in real life. In line with Ohara (1999), we operationalized politeness as formality (or lack thereof) of the addressee: for each language, images of an older-looking female and male in business-like attire (formal addressees) and images of a younger-looking female and male in school uniforms (informal addressees) were chosen, totaling four images for each language.^3^ The (in)formality of the addressees was judged by the three authors and confirmed by a small feasibility test carried out in London (UK).^4^ For each formality level, we chose one image that would be typically assumed to be of a female and one image that would be typically assumed to be of a male.^5^ All participants saw the same combinations of sentence token and addressee (Appendix C). Presentation order was fully randomized.

### 2.4 Procedure

Recordings were conducted at the Phonetics Laboratory of Queen Mary University of London (UK) and Sophia University (Tokyo, Japan). To avoid drop-outs, bilinguals completed the study in both of their languages on the same day, with a 30-minute break between the two sessions to account for language modes (Grosjean, 2010). Languages were counterbalanced across bilinguals to control for learning effects and fatigue (Graham, 2015).

**Figure 1.**
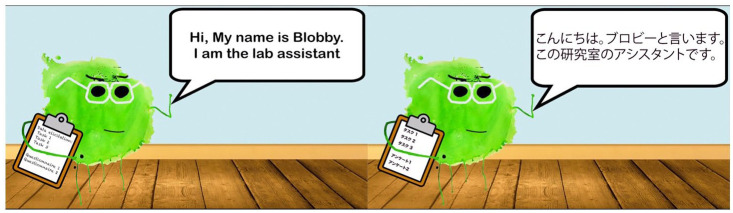
Gender neutral animation character, English version on the left and Japanese on the right.

In both locations, the first author welcomed bilinguals in English and accompanied them to the recording room, where they saw a simple interface built in PsychoPy 1.85.3 (Peirce, 2007). To minimize interference from the investigator (Carrie & Drummond, 2017), phonetic imitation (Adank et al., 2013), and/or gender interactions (Biemans, 1998), written instructions were provided with short custom-made animated clips (created in Adobe Character Animator; Adobe, 2017) featuring a fantasy character named Blobby designed to be as gender-neutral as possible (Figure 1). The first author created the script of the animation in English, which was then translated into Japanese following the procedure outlined in Brislin (1970).

Blobby instructed participants to read the sentences aloud without changing their content in any way, addressed to the image of the person presented to them on the monitor (see section 2.3). If they thought they had made an error, participants had to repeat the entire sentence. A short practice trial with different sentences and addressees preceded the main task. Practice trial data were not included in the analysis. Participants filled in the relevant gender questionnaire at the end of each session of the data collection.

The recording chain was a Røde NT1-A condenser microphone (cardioid polar pattern) and a Steinberg UR22 audio interface (microphone preamp and analogue-to-digital converter). All audio was recorded direct-to-disk on a MacBook Pro located outside the testing booth, at a sample rate of 44.1 kHz, 16-bit.

### 2.5 Acoustic analysis

The phonetic variables analyzed were mean F0 in Hertz (Hz) and 80% span in semitones (ST) (de Leeuw, 2020; Ordin & Mennen, 2017). They represent level and span, the two quasi-dependent dimensions along which pitch range varies (Ladd, 2008).

The first author carried out the acoustic analysis in Praat (Boersma & Weenink, 2016), using the autocorrelation method for pitch tracking. Pitch floor and ceiling were kept as recommended in the Praat manual at 100–500 Hz for females and 75–300 Hz for males. Recordings were visually and auditorily inspected in 5/10-second intervals to check for octave jumps and/or doubling, and sections of creaky voice were removed from the analysis (de Leeuw, 2019; Passoni et al., 2019). Thereafter, pitch range measurements were extracted with a customized script over the whole utterance.

### 2.6 Statistical analysis

All data were analyzed using R version 3.6 (R Core Team, 2019) with the R packages *lme4* package (Bates et al., 2015) and *lmerTest* (Kuznetsova et al., 2017). All plots were created using the *ggplot2* package (Wickham, 2016). We performed two series of linear mixed-effects models (LMERs) with treatment contrasts. Maximal models (i.e., models with all main effects and interactions) were automatically stepped-down using the *step* function from *lmerTest* (Kuznetsova et al., 2017) to achieve a best-fit model. The significance of each best-fit model’s coefficient was estimated using the Satterthwaite method from *lmerTest* (Kuznetsova et al., 2017) and significance level was set at *p* **<** .05. If results revealed significant interactions between predictors, any potential main effect was not expanded upon because main effects are uninterpretable in the case of a significant interaction (see Winter & Grawunder, 2012). Results were reported with standard errors (*SE*s). Residual plots were visually inspected to detect any obvious deviation from normality and homoscedasticity. Post hoc analyses were run using the *emmeans* package (Lenth & Love, 2017) with levels of significance Bonferroni-adjusted for pairwise comparisons.

## 3 Results

Results on cross-language variation (RQ1) are presented in section 3.1 and on expression of social meaning in the two languages of the bilinguals (RQ2) in section 3.2. Due to space limitations, post hoc tables are in Appendix D.

### 3.1 Cross-language variation

To investigate whether pitch range significantly differed between the two languages of the two groups of bilingual females and males (RQ1), we built two models (i.e., one for pitch level and one for pitch span) with language (English vs. Japanese), sex of the participant (female vs. male), and testing location (London vs. Tokyo) as fixed independent factors, and participant as random intercept. Models including by-token random intercepts as well as by-participant and by-token random slope were tested but failed to converge.

#### 3.1.1 Pitch level

Table 3 reports model parameters for the best-fit model for mean F0. There was a significant main effect of sex of the participant (*p* **<** .0001) and a significant interaction between language and testing location (*p* = .001).

**Table 3.** Results for the Best-Fit Model for Mean F0 Variation between the Two Languages of the Bilinguals.

Fixed effects	Estimate	Standard error	*t* value	*p* value
Intercept (language = English, sex of the participant = female, testing location = London)	237.307	4.087	58.06	<.0001
Language = Japanese	−14.616	1.045	−13.98	<.0001
Sex of the participant = male	−102.431	5.361	−19.11	*<.0001*
Testing location = Tokyo	−3.954	4.943	−0.80	.428
Language = Japanese: testing location = Tokyo	6.06	1.427	4.21	.001

*Note. N* = 1,312; random intercepts = participant (41); log likelihood = −5,293.6; conditional *R*^2^ = 0.94; significance level *p* **<** .05.

**Figure 2.**
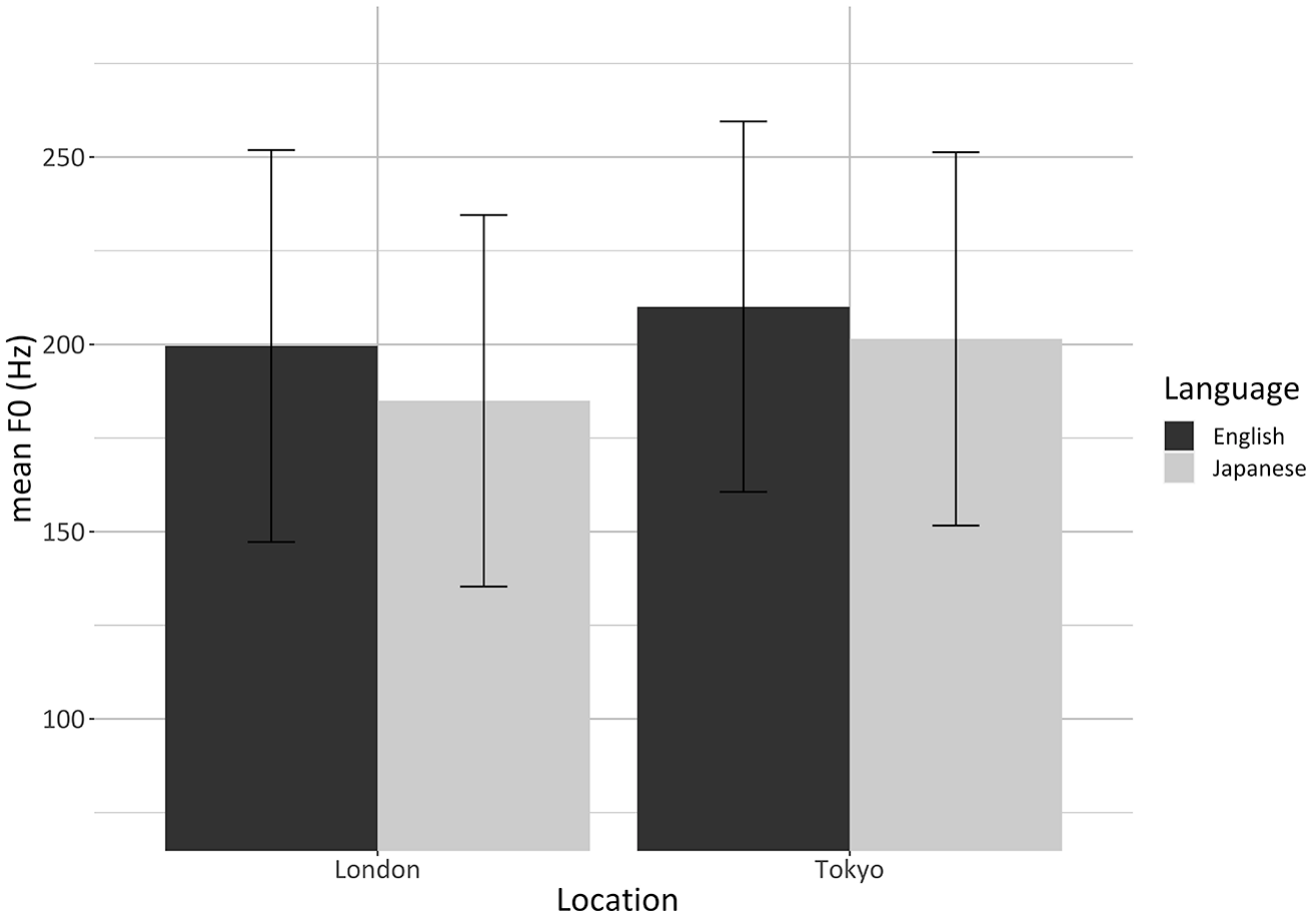
Barplots showing the significant interaction between language and testing location for mean F0. Values are averages for each participant; error bars represent standard errors.

Figure 2 illustrates the significant interaction between language and testing location. Post hoc analyses indicated that, irrespective of their sex, bilinguals produced English with a higher mean F0 than Japanese. This held true in both London and Tokyo; however, the magnitude of the effect was larger in London. Specifically, (1) the English mean F0 of the bilinguals tested in London was on average 14.6 ± 1.0 Hz higher than their Japanese mean F0 (*p* **<** .0001), whereas (2) the English mean F0 of the bilinguals tested in Tokyo was on average 8.16 ± 0.9 Hz higher than their Japanese mean F0 (*p* **<** .0001) (Appendix D, Table D1).

**Figure 3.**
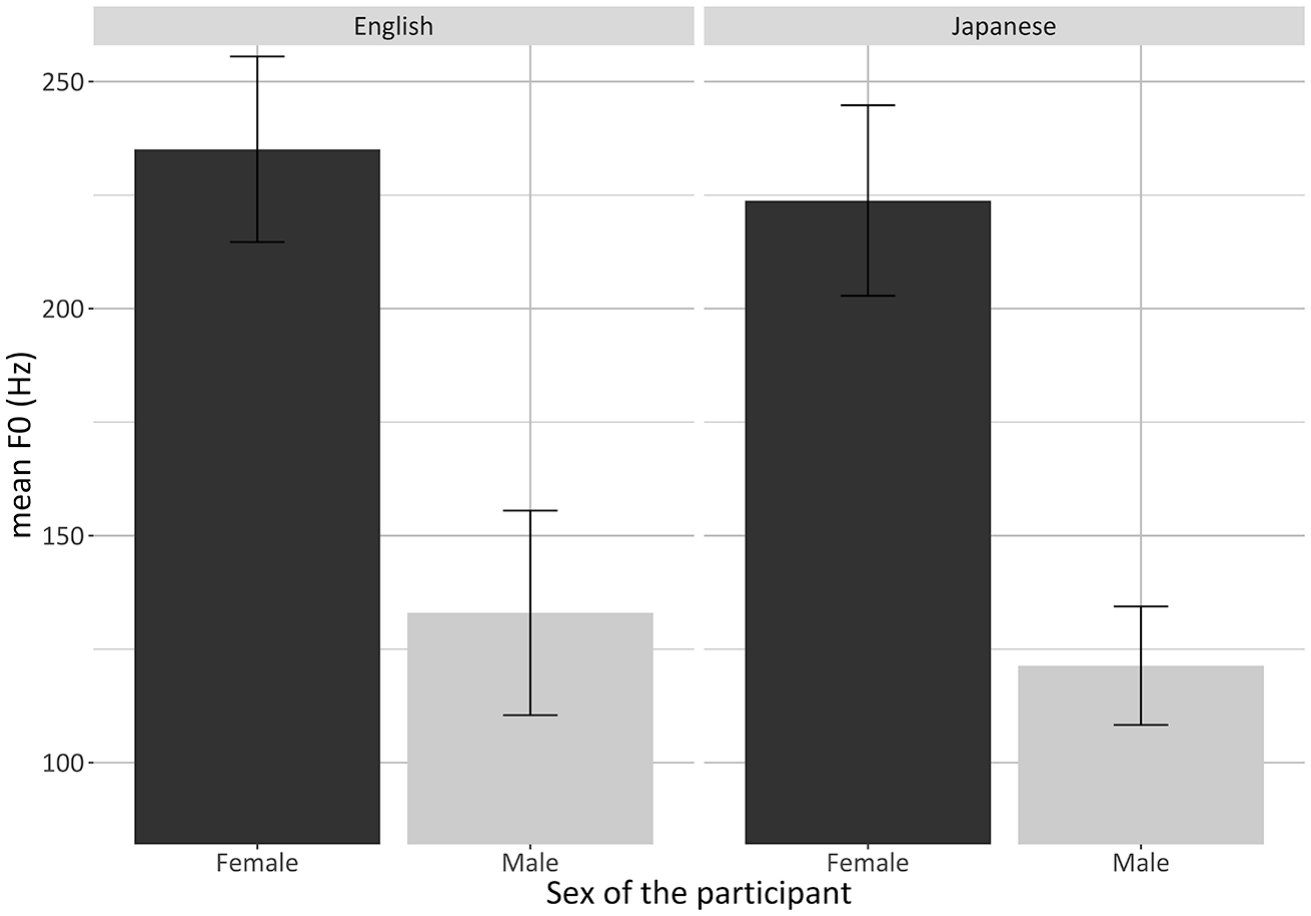
Barplots showing the significant main effect of sex of the participant for the mean F0 of both languages of the bilinguals separately. Values are averages for each participant; error bars represent standard errors.

Unsurprisingly, the mean F0 of the females was on average 102.4 ± 5.3 Hz higher than the mean F0 of the male bilinguals (*p* **<** .0001) (Figure 3).

#### 3.1.2 Pitch span

**Figure 4.**
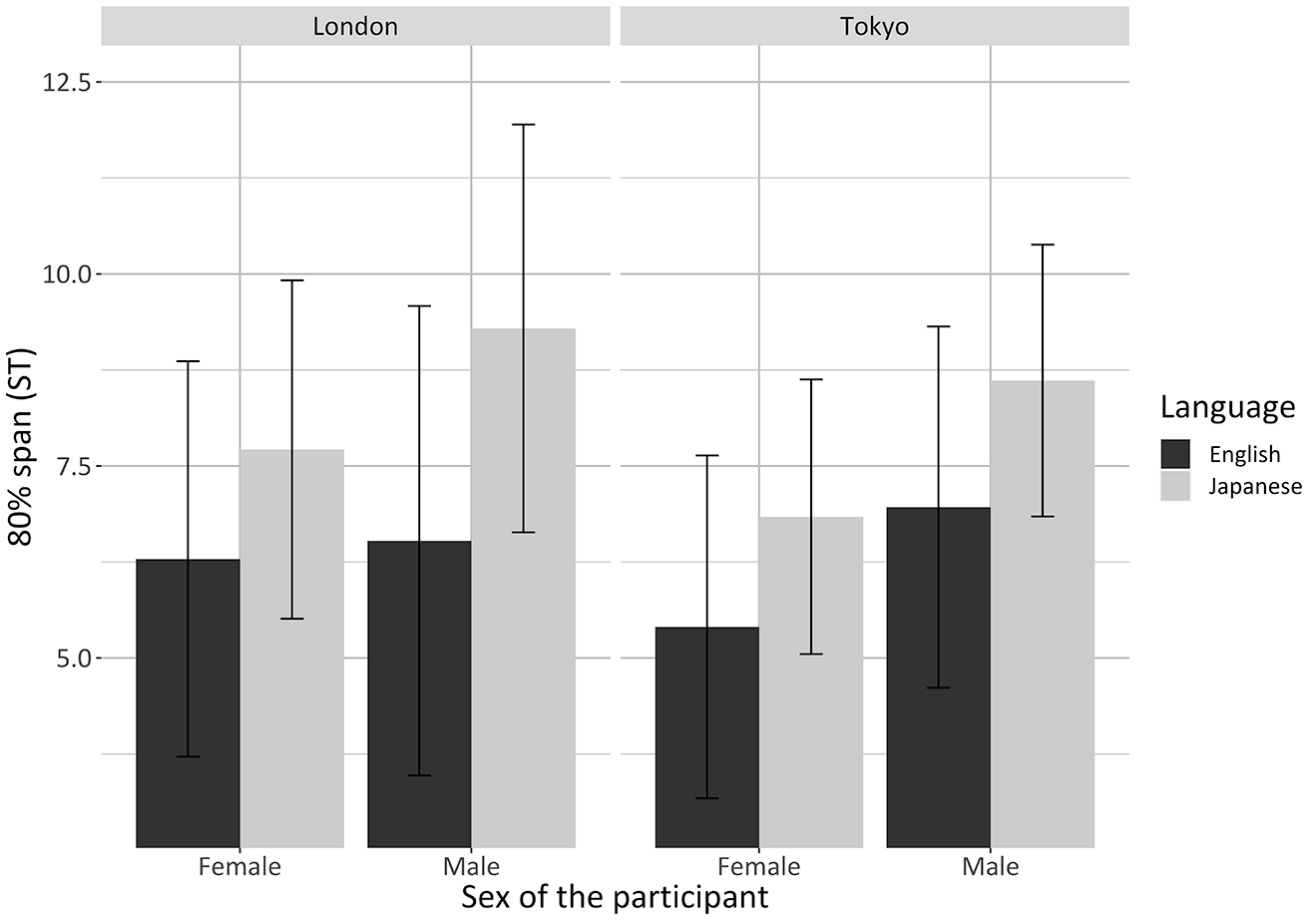
Barplots showing the significant interaction between language, sex of the participant, and testing location for 80% span. Values are averages for each participant; error bars represent standard errors.

Table 4 reports model parameters for the best-fit model for 80% span. There was a significant interaction between language, sex of the participant, and testing location (*p* = .009) (Figure 4).

**Table 4.** Linear Mixed-Effects Regression Results for 80% Span Variation Between the Two Languages of the Bilinguals.

Fixed effects	Estimate	Standard error	*t* value	*p* value
Intercept (language = English, sex of the participant = female, testing location = London)	6.28	0.44	14.24	<.0001
Language = Japanese	1.42	0.18	7.90	<.0001
Sex of the participant = male	0.23	0.73	0.33	.746
testing location = Tokyo	–0.88	0.58	–1.53	.132
Language = Japanese: sex of the participant = male	1.33	0.30	4.51	<.0001
Language = Japanese: testing location = Tokyo	0.01	0.24	0.04	.968
Sex of the participant = male: testing location = Tokyo	1.32	1.07	1.24	.221
Language = Japanese: sex of the participant = male: location = Tokyo	–1.15	0.44	–2.58	.009

*Note. N* = 1,312; random intercepts = participant (41); log likelihood = −2,673.15; conditional *R*^2^ = 0.52; significance level *p* **<** .05.

Post hoc analyses indicated that bilinguals produced English with a significantly narrower pitch span than Japanese (*p* < .0001). This was again valid for both females and males and in both testing locations; however, the magnitude of the effect was at its largest for the males tested in London. Specifically, (1) the English 80% span of the male bilinguals tested in London was on average 2.76 ± 0.2 ST narrower than their Japanese span (*p* < .0001), whereas (2) the English 80% span of the male bilinguals tested in Tokyo was on average 1.65 ± 0.3 ST narrower than their Japanese span (*p* < .0001). In addition, (3) the English 80% span of the female bilinguals in London and Tokyo was on average 1.43 ± 0.2 ST narrower than their Japanese 80% span (*p* < .0001) (Appendix D, Table D2).

Summarizing, with regard to RQ1, irrespective of the sex of the participant and testing location, results indicated that there were significant differences between the pitch range of the English and the Japanese of the bilinguals. Unexpectedly, English mean F0 was significantly higher than Japanese mean F0, whereas English 80% span was significantly narrower than Japanese 80% span.

### 3.2 Expression of social meaning in English and Japanese

To investigate potential differences in how social meaning was expressed through pitch range, we carried out a second series of mixed-effects models on each language separately. Significance levels for the analyses below were Bonferroni-adjusted to *p* **<** .025 (Mundfrom et al., 2006). Maximal models included fixed effects of sex of the participant, individual gender identity,^6^ formality of the imagined addressee, and sex of the addressee. As before, participant was entered as random intercept and the pitch measurement of interest (mean F0 and 80% span) as dependent variables. Again, models including by-token random intercepts, by-participant and by-token random slope were tested but failed to converge.

#### 3.2.1 Pitch level

Table 5 reports model parameters for the best-fit model for English pitch level. There was a significant interaction between individual gender scores (as operationalized by the BSRI-short—see section 2.2) and sex of the participant (*p* = .016 and *p* = .004).

**Table 5.** Linear Mixed-Effects Regression Results for the Bilinguals’ English Mean F0.

Fixed effects	Estimate	Standard error	*t* value	*p* value
Intercept (sex of the participant = female)	270.21	17.212	15.699	<.0001
Sex of the participant = male	−121.43	25.091	−4.84	<.0001
Femininity BSRI-short	1.88	3.807	0.494	.624
Masculinity BSRI-short	−10.87	3.667	−2.965	.005
Sex of the participant = male: femininity BSRI-short	−13.76	5.486	−2.509	.016
Sex of the participant = male: masculinity BSRI-short	18.72	4.923	3.804	.004

*Note. N* = 656; random intercepts = participant (41); log likelihood = −1,229.01; conditional *R*^2^ = 0.93, significance level *p* **<** .025 (Bonferroni-corrected).

**Figure 5.**
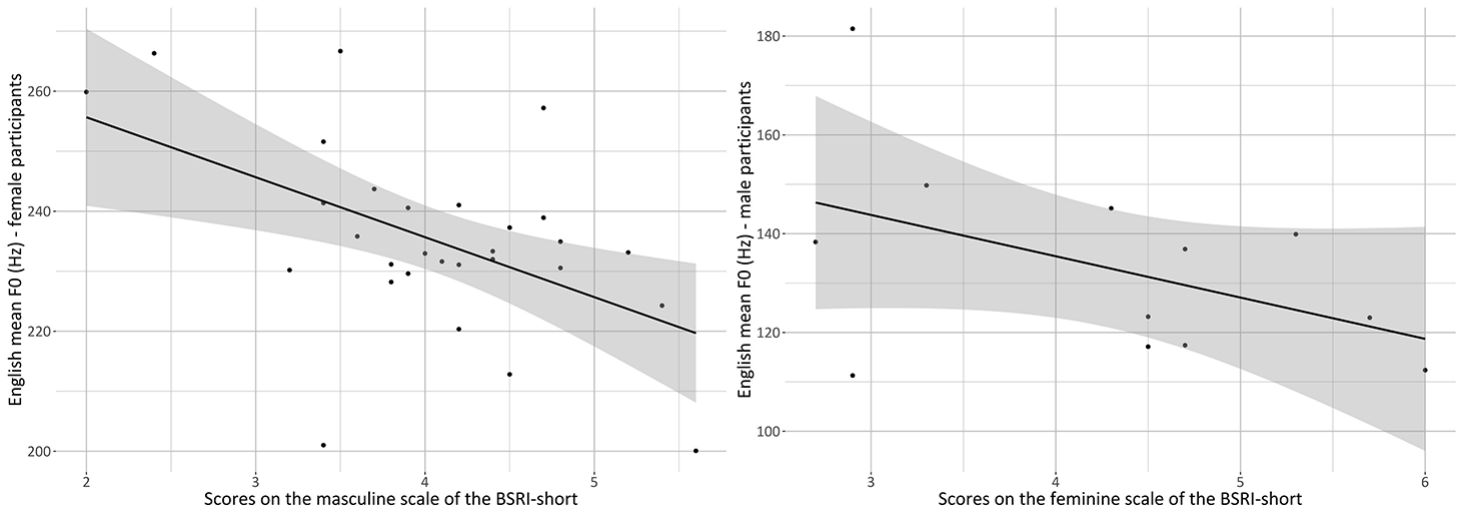
Scatterplots showing the significant relationship between scores on the masculinity scale of the BSRI-short and mean F0 in the English of the females (left panel) and between scores on the femininity scale of the BSRI-short and mean F0 in the English of the males (right panel). Values are averages for each participant.

Post hoc analysis indicated that higher self-attribution of masculine traits on the BSRI-short patterned with lower pitch level in the English of the females (*p* = .008) (Figure 5, left panel and Appendix D, Table D3). This corresponds to popular stereotypes of gender and pitch in English. For males, however, a higher self-attribution of feminine traits surprisingly patterned with lower pitch level in their English (*p* = .007) (Figure 5, right panel and Appendix D, Table D4). This pattern appears counter-stereotypical, indicating that men who reported a stronger affiliation with feminine gender norms showed lower pitch levels than those with weaker ties to English feminine gender norms. Therefore, results indicated that more masculine females and more feminine males produced the lowest mean F0s in their L2.

**Figure 6.**
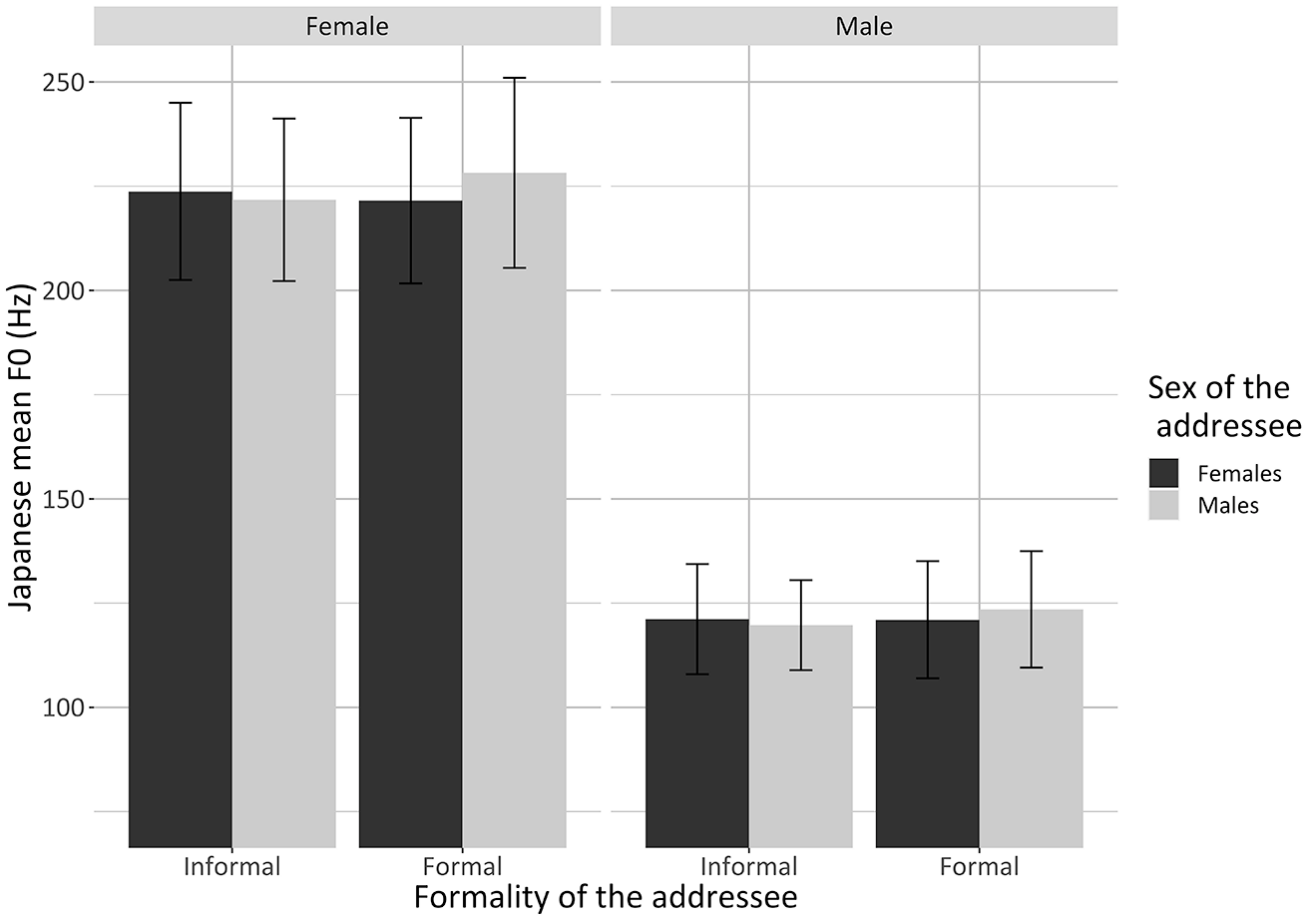
Barplots showing the significant two-way interaction between formality of the addressee and sex of the addressee for the mean F0 in the Japanese of the bilingual females and males separately. Values are averages for each participant; error bars represent standard errors.

While in English formality and sex of the addressee were not shown to have a significant effect on mean F0, in Japanese there was a significant interaction between formality and sex of addressee (*p* = .001), as well as a significant main effect of sex of the participant (*p* **<** .0001) (Table 6). Post hoc comparisons revealed that the Japanese formal-looking male elicited (1) a mean F0 5.4 ± 1 Hz higher than the formal female (*p* **<** .0001) (Figure 6) and (2) a mean F0 5.7 ± 1 Hz higher than the Japanese informal-looking male (*p* **<** .0001) (Figure 6). No significant differences were detected between the mean F0 elicited by the two Japanese female addressees (formal vs. informal) and between the two informal-looking addressees (female vs. male) (Appendix D, Table D5). Notably, these results were confirmed for female and male bilinguals (Figure 6). Unlike for English, Japanese pitch level did not appear to be affected by individual gender identity, as operationalized by the JGRI questionnaire.

**Table 6.** Linear Mixed-Effects Regression Results for the Bilinguals’ Japanese Mean F0.

Fixed effects	Estimate	Standard error	*t* value	*p* value
Intercept (sex of the participant = female, formality of the addressee = informal, sex of the addressee = female)	223.69	3.09	72.31	<.0001
Sex of the participant = male	–102.44	5.59	–18.34	*<.0001*
Formality of the addressee = formal	–1.60	1.08	–1.47	.114
Sex of the addressee = male	–1.83	1.08	–1.69	.009
Formality of the addressee = formal: sex of the addressee = male	7.28	1.53	4.74	.001

*Note. N* = 656; random intercepts = participant (41); log likelihood = −2,506.9; conditional *R*^2^ = 0.96; significance level *p* **<** .025 (Bonferroni-corrected).

Summarizing, with regard to RQ2, the analysis for pitch level indicated that in English, but not in Japanese, individual gender identity patterned with interpersonal variation among the bilinguals in their mean F0. Somewhat expectedly, for females, higher attribution of masculine traits on the BSRI-short patterned with lower mean F0s; surprisingly, for males, higher attribution of feminine traits in the BSRI-short patterned with lower mean F0s. Continuing, in Japanese, but not in English, the mean F0 of the female and male bilinguals was affected by formality and sex of the imagined addressee. Specifically, the formal-looking male elicited a significantly higher mean F0 than the formal-looking female and the informal-looking male. No significant differences were evidenced between the mean F0 elicited by the two female addressees and between the informal-looking addressees.

#### 3.2.2 Pitch span

This section explores whether expression of social meaning impacted variation in the pitch span of the two languages of the bilinguals differently.

For English, the best-fit model revealed that none of the predictors under consideration explained variation in the pitch span of the bilinguals.

**Figure 7.**
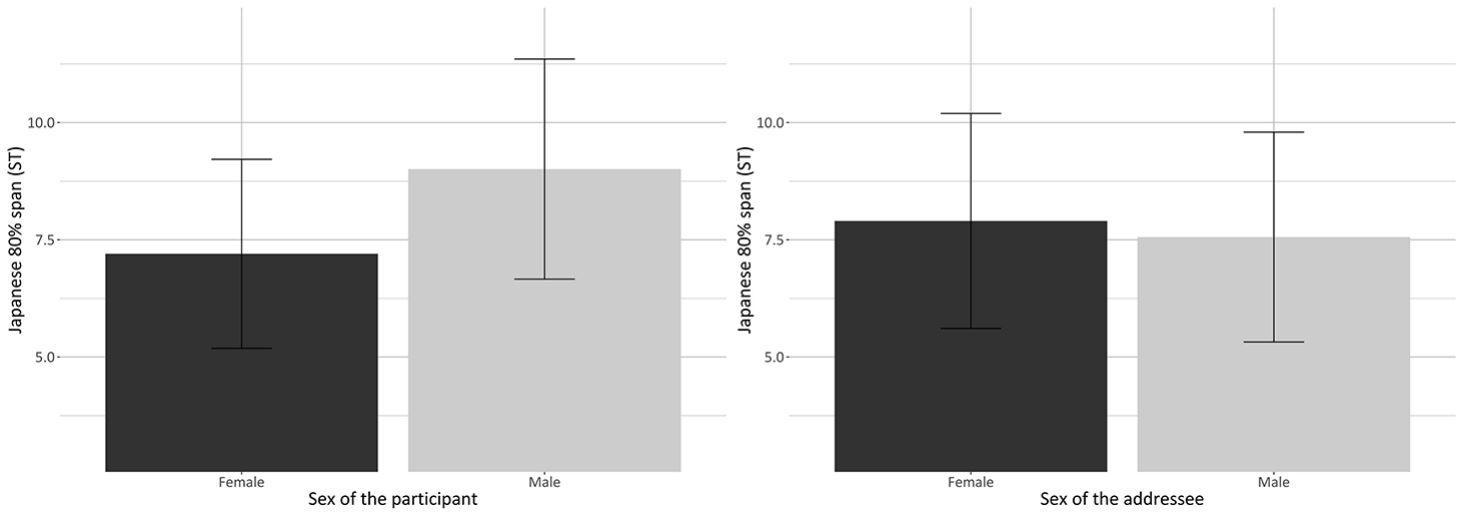
Barplots showing the significant main effect of sex of the participant for the 80% span of the Japanese of the bilinguals (left panel) and the significant main effect of sex of the addressee for the 80% span of the Japanese of the bilinguals (right panel). Values are averages for each participant; error bars represent standard errors.

Table 7 reports model parameters for the best-fit model for Japanese 80% span. There was a significant main effect of sex of the addressee (*p* = .003) and sex of the participant (*p* = .002) (Figure 7).

**Table 7.** Linear Mixed-Effects Regression Results for the Bilinguals’ Japanese 80% Span.

Fixed effects	Estimate	Standard error	*t* value	*p* value
Intercept (sex of the participant = female, sex of the addressee = female)	7.37	0.30	24.30	<.0001
Sex of the participant = male	1.80	0.55	3.28	.002
Sex of the addressee = male	−0.34	0.11	−3.01	.003

*Note. N* = 656; random intercepts = participant (41); log likelihood = −1,237.4; conditional *R*^2^ = 0.59; all interactions *p* **>** .025 (Bonferroni-corrected).

The pitch span of the male bilinguals was 1.8 ± 0.5 ST wider than that of the female bilinguals in Japanese (*p* = .002) (Figure 7, left panel). In addition, female addressees elicited a pitch span 0.3 ± 0.1 ST wider than male addressees (*p* = .003) in the Japanese of the bilinguals (Figure 7, right panel).

Summarizing, with regard to RQ2, none of the variables considered explained variation in the bilinguals’ English pitch span. Alternatively, in Japanese, sex of the addressee and sex of the participant affected the pitch span of the female and male bilinguals. Specifically, female addressees elicited a larger pitch span than male addressees and male bilinguals produced Japanese with a wider span than female bilinguals.

## 4 Discussion

The present study sought to find out whether female and male Japanese-English sequential bilinguals produced pitch range differently in their two languages (RQ1) and whether potential within-language variation could be explained by the social meaning(s) that each of these languages associate with pitch level and pitch span (RQ2).

With regard to RQ1, surprisingly, in London *and* Tokyo, female *and* male bilinguals produced English with a significantly higher pitch level than Japanese. This was not in line with Hypothesis 1a, nor with previous work which has reported that Japanese is produced, at least by female bilinguals, with a higher mean F0 than English (Graham, 2015; Loveday, 1981; Ohara, 1992, 1999). The contrast of the current pitch-level results with findings of previous related work on Japanese-English bilinguals was unexpected and may be tentatively explained in terms of confidence. Despite reporting an overall fairly high level of L2 proficiency (on average 7/10), all bilinguals indicated that Japanese was their dominant language, that is, the language they felt most confident speaking. Prior to data collection, participants were assured that their L1 and L2 proficiencies were not the focus of the study. Nonetheless, insecurities when speaking English—which might also be linked to feeling less aware of the sociocultural norms of the English language—might have led to an increase in stress during the English task. Higher levels of stress have been reported to lead to a higher pitch level (Scherer, 2003), due to increased tension in the laryngeal structures (Järvinen et al., 2013). In line with this, Gussenhoven (2002) claimed that a higher pitch level conveys uncertainty and a lower pitch certainty. Likewise, research on vocal cues of confidence has indicated that voices perceived as doubtful are marked by a higher pitch level (Jiang & Pell, 2017). Interestingly, during the debriefing, one of the female bilinguals tested in London remarked that her voice is higher in English than Japanese because, despite having lived in the United Kingdom for 10 years and having been married to a British man for 8 years, when she speaks English she does not feel as confident as she does in Japanese and is worried that she might make mistakes.

In line with H1b and Graham (2015), pitch span was wider in the Japanese than the English of the female *and* male bilinguals in London *and* Tokyo. Interestingly, male bilinguals produced Japanese with a wider pitch span than female bilinguals did (Guillemot & Sano, 2020). This adds to the body of evidence that suggests that Japanese males do not necessarily only speak with a monotonous voice (see, for example, Sturtz Sreetharan, 2004).

It was surprising that the above-mentioned differences between Japanese and English in the bilinguals were found in both London and Tokyo, although the differences in London were greater than in Tokyo. Previous research has reported that L1 prosodic variables are susceptible to attrition effects potentially due to L2 exposure (de Leeuw, 2019; de Leeuw et al., 2012; Mennen, 2004). In the current study, this may have been, for example, substantiated as the difference between the higher English F0 and the lower Japanese F0 was greater in the London bilinguals than in the Tokyo bilinguals. Essentially, although the same effect was found in both groups of bilinguals, the magnitude of the effect was greater in the London bilinguals than in the Tokyo bilinguals. Future publications arising from this research will compare the bilingual groups with functional monolingual groups to more adequately assess whether there were differences in L2 acquisition and L1 attrition.

Continuing with RQ2, in line with H2a and H2b, variation in the imagined addressee did not affect the pitch range of the English of the bilinguals. Interestingly, individual gender identity (H2f) explained variation in the English mean F0, but not 80% span, of the bilinguals and this was dependent on the sex of the participant. Specifically, results indicated that a higher self-attribution of masculine traits on the BSRI-short patterned with lower mean F0s among the female bilinguals and, surprisingly, a higher self-attribution of feminine traits patterned with lower mean F0s among the males. In other words, more masculine females and more feminine males produced the lowest mean F0s in English. The link between enhanced masculinity and lower mean F0s in female speakers has been hypothesized elsewhere (Biemans & Van Bezooijen, 1996). Our analysis provided quantitative evidence that, among these female bilinguals, a more masculine gender identity is correlated with lower mean F0s in their English. On the contrary, the link between enhanced femininity and lower mean F0s in the English of the male bilinguals appears counterintuitive, as a higher mean F0 is normally assumed to index a more feminine gender identity (Biemans & Van Bezooijen, 1996). Eckert’s (2008) notion of “indexical field” may be of help in explaining this result. She maintains that the meaning of a (phonetic) variant is not fixed but is distributed over a field of “ideologically related meanings, any one of which may be activated in the situated use of the specific variable” (2008, p. 453). Thus, the use of a variable does not necessarily activate a unique and predetermined indexical meaning, rather a variety of ideologically linked meanings. Applying this model to the case of mean F0 in the English of the bilinguals, we propose that the mapping between form and function happens at the first indexical order, that is, at the level of membership to a population (Silverstein, 2003), in this case at the level of signaling membership to the population women. The mapping between form and meaning, however, happens at the second lexical order, that is the level at which the linguistic form becomes a stylistic marker (Silverstein, 2003), in this case at the level of signaling politeness (i.e., a trait that is traditionally attributed to the population women). We suggest therefore that *lower mean F0* activated different indexical meanings in the English of the bilingual females and males of the present sample. Specifically, in the speech of the bilingual females, it activated the first-order indexical meaning of “decreased femininity” (or “increased masculinity”), whereas in the speech of the bilingual males, it activated the second-order indexical meaning of “decreased politeness”. Importantly, politeness is linked to friendliness in English (Pizziconi, 2007), thus it is not dependent on hierarchy, which could explain why no effect of formality of the imagined addressee was detected on the bilinguals’ English.

Variation in the imagined addressee also affected the pitch range of the Japanese of the bilinguals. Pitch-level findings were partially in line with previous work (Ohara, 1999, 2004) and H2c. We found that mean F0 was affected by both formality and sex of the addressee. Specifically, the formal male elicited a significantly higher mean F0 than the formal female, but this was not replicated across the informal addressees. In addition, the formal male elicited a significantly higher mean F0 than the informal male addressee, but this was not the case for the two female addressees. To our knowledge, this is the first study to report that formality *and* sex of the addressee may have a combined effect on Japanese pitch level. With regard to pitch span, we found that it only patterned with variation in the sex of the addressee (H2f). Specifically, female addressees elicited a larger pitch span than male addressees in the Japanese of the bilinguals; this, to our knowledge, has also not been reported elsewhere. We tentatively suggest that, in Japanese, expression of politeness via pitch range modulation may be dependent not only on the status but also on the sex of the addressee.

Its surprising that our Japanese pitch level results did not appear dependent on (1) whether the speaker was female or male, as the previous literature has indicated that pitch range manipulation to signal politeness is peculiar to JWL (Ohara, 1999, 2004), nor on (2) the bilinguals’ Japanese gender identity scores. Methodological differences may be of help in explaining the discrepancy between the current work and the previous literature: Ohara (1999) analyzed spontaneous speech, whereas we analyzed read speech. In Japanese, politeness is marked lexically, grammatically and phonetically (Sherr-Ziarko, 2019). It may be that in the present study, where participants could only manipulate the phonetic dimension of their speech, as it is a reading task, males did not hesitate to do so. With regard to the bilinguals’ Japanese gender identity, whether the lack of pattern is due to specific characteristic**s** of the present speakers is unclear. Previous research has reported that parallels between F0 patterns and gender identity are an indication of sociocultural influence on pitch range (Moore, 1995; Weirich & Simpson, 2018); therefore, assuming English society to be more gender-egalitarian than the Japanese society and considering gender-specific norms to be more prominent in Japanese than English (see also Ohara, 2019), we speculate that the bilinguals may have felt “freer” to use their pitch range to express their individual gender identity in their L2, as opposed to the normative gender identity they may be expected to express in their L1.

Despite certain limitations, such as an unequal number of female and male participants, an examination of read speech over spontaneous speech and the lack of comparisons with monolingual speakers, we maintain that our findings are noteworthy both for the fields of bilingualism and sociophonetics. Our results add to existing evidence that sequential bilinguals realize pitch range differently in their two languages and that these differences may be more extreme in an L2 environment. The finding that the mean F0 behavior of female bilinguals is compatible with the expression of individual gender identity in English and politeness norms in Japanese is in line with previous work suggesting that female Japanese-English bilinguals appear under social pressure to perform a normative female gender when speaking Japanese, but not English (Ohara, 1999). The finding that also male bilinguals manipulate mean F0 to express social meaning in their two languages is novel (Loveday, 1981; Ohara, 1999); however, in line with relatively recent work indicating that Japanese males may use pitch range dynamically, if needed (Hiramoto, 2010; Ofuka et al., 2000). More generally, our findings confirm that female and male Japanese-English sequential bilinguals living in London and Tokyo produce pitch range differently in their two languages and that the pitch range differences between their two languages appear to be greater in London than in Tokyo. Moreover, we provide evidence that bilinguals manipulate the pitch range of their two languages to express nuanced language specific social meaning(s); that is, expression of individual gender identity in English (but not Japanese) and expression of politeness in Japanese (but not English). As such, we believe that our research indicates that bilinguals are active agents in their language use and choices (Hansen Edwards, 2008) and that, similarly to monolinguals, use phonetic variation to make social moves and express identities.

## Supplemental Material

sj-xlsx-1-las-10.1177_00238309221105210 – Supplemental material for Bilinguals Produce Pitch Range Differently in Their Two Languages to Convey Social Meaning Click here for additional data file. Supplemental material, sj-xlsx-1-las-10.1177_00238309221105210 for Bilinguals Produce Pitch Range Differently in Their Two Languages to Convey Social Meaning by Elisa Passoni, Esther de Leeuw and Erez Levon in Language and Speech

## Appendix A

### Background questionnaire

**Table A1.** Analysis of Variance for Biological Age.

Variable	*df*	Sum of squares	Mean square	*F*	*p* value	η^2^
Location	1	348.2	348.2	13.9	.001	.273
Sex of the participant	1	17.1	17.1	0.7	.413	.012
Location: sex of the participant	1	0.0	0.0	0.0	.995	.001
Residuals	37	386.3	10.44			

**Table A2.** Analysis of Variance for Age of Acquisition.

Variable	*df*	Sum of squares	Mean square	*F*	*p* value	η^2^
Location	1	32.6	32.6	3.1	.085	.078
Sex of the participant	1	13.2	13.2	1.2	.267	.033
Location: sex of the participant	1	11.6	11.6	1.1	.299	.029
Residuals	37	386.3	10.4			

**Table A3.** Analysis of Variance for L2 Proficiency.

Variable	*df*	Sum of squares	Mean square	*F*	*p* value	η^2^
Location	1	2.17	2.17	0.81	.374	.021
Sex of the participant	1	8.34	8.34	3.11	.084	.077
Location: sex of the participant	1	0.07	0.07	.027	.870	.001
Residuals	37	99.3	2.684			

## Appendix B

### Gender questionnaires

**Table B1.** Average Ratings (Mean and Standard Deviation) for Each Trait of the Femininity Scale of the BSRI-Short for Female and Male Bilinguals Separately.

BSRI-short femininity scale	Mean scores, females	Mean scores, males
Affectionate	4.6 (1.6)	4.4 (1.7)
Sympathetic	4.8 (1.7)	4.5 (1.8)
Sensitive to others’ needs	5.2 (1.4)	5.1 (1.5)
Understanding	5.0 (1.3)	4.8 (1.4)
Compassionate	4.4 (1.2)	4.4 (1.2)
Eager to soothe feelings	4.8 (1.2)	4.8 (1.2)
Warm	4.5 (1.2)	4.5 (1.3)
Tender	4.1 (1.3)	4.3 (1.3)
Love children	4.6 (2.0)	4.4 (1.9)
Gentle	4.5 (1.3)	4.3 (1.4)

BSRI-short: Bem Sex Role Inventory–short.

**Table B2.** Analysis of Variance for the Femininity Scores on the BSRI-Short.

Variable	*Df*	Sum of squares	Mean square	*F*	*p* value	η^2^
Sex of the participant	1	2.07	2.07	2.66	.111	.060
Residuals	39	30.38	0.779			

*Note*. BSRI-short: Bem Sex Role Inventory–short.

**Table B3.** Average Ratings (Mean and Standard Deviation) for Each Trait of the Masculinity Scale of the BSRI-Short for Female and Male Bilinguals Separately.

BSRI-short masculinity scale	Mean scores, females	Mean scores, males
Defend my own beliefs	4.9 (1.4)	5.0 (1.4)
Independent	4.8 (1.5)	4.8 (1.6)
Assertive	4.1 (1.6)	4.2 (1.6)
Strong personality	4.5 (1.6)	4.8 (1.7)
Forceful	3.7 (1.5)	4.1 (1.3)
Have leadership abilities	4.0 (1.7)	4.1 (1.8)
Willing to take risks	4.1 (1.6)	3.9 (1.7)
Dominant	3.8 (1.3)	3.8 (1.2)
Willing to take a stand	4.3 (1.4)	4.0 (1.5)
Aggressive	3.6 (1.6)	3.6 (1.6)

*Note.* BSRI-short: Bem Sex Role Inventory–short.

**Table B4.** Analysis of Variance for the Masculinity Scores on the BSRI-Short.

Variable	*df*	Sum of squares	Mean square	*F*	*p* value	η^2^
Sex of the participant	1	1.53	1.53	1.58	.216	.038
Residuals	39	38.81	0.96			

*Note*. BSRI-short: Bem Sex Role Inventory–short.

**Table B5.** Average Ratings (Mean and Standard Deviation) for Each Trait of the Femininity Scale of the JGRI for Female and Male Bilinguals Separately.

JGRI femininity scale	Mean scores, females	Mean scores, males
Innocent	4.1 (1.7)	4.2 (1.8)
Graceful	5.0 (1.5)	5.0 (1.6)
Affectionate	5.2 (1.5)	5.3 (1.5)
Have charm	4.8 (1.4)	5.0 (1.4)
Attentive to the needs of others	4.0 (1.7)	4.1 (1.7)
Polite	4.7 (1.5)	4.9 (1.6)
Calm	4.7 (1.5)	4.7 (1.4)
Love children	4.0 (1.4)	3.9 (1.6)
Like to care for others	4.1 (1.4)	4.1 (1.4)
Have neat habits	4.4 (1.5)	4.6 (1.4)

*Note*. JGRI: Japanese Gender Role Index.

**Table B6.** Analysis of Variance for the Femininity Scores on the JGRI.

Variable	*df*	Sum of squares	Mean square	*F*	*p* value	η^2^
Sex of the participant	1	0.08	0.08	0.067	.797	.000
Residuals	39	43.64	1.119			

*Note.* JGRI: Japanese Gender Role Index.

**Table B7.** Average Ratings (Mean and Standard Deviation) for Each Trait of the Masculinity Scale of the JGRI for Female and Male Bilinguals Separately.

JGRI masculinity scale	Mean scores, females	Mean scores, males
Have a leadership ability	4.3 (1.6)	4.3 (1.7)
Strong willed	3.4 (1.6)	3.3 (1.8)
Ability to implement action of one’s own accord	4.6 (1.5)	4.7 (1.7)
Have a broad perspective	3.2 (1.6)	3.5 (1.7)
Ability to bring others together	4.7 (1.6)	4.8 (1.6)
Have guts	4.0 (1.7)	4.1 (1.8)
Become self-supportive	4.0 (1.7)	3.7 (1.7)
Persuasive	4.4 (2.0)	4.2 (2.0)
Relied on by others	4.2 (1.6)	4.2 (1.7)
Upstanding	4.3 (1.6)	4.2 (1.7)

*Note*. JGRI: Japanese Gender Role Index.

**Table B8.** Analysis of Variance for the Masculinity Scores on the JGRI.

Variable	*df*	Sum of squares	Mean square	*F*	*p* value	η^2^
Sex of the participant	1	3.26	3.26	3.96	.054	.010
Residuals	39	32.08	0.82			

*Note*. JGRI: Japanese Gender Role Index.

## Appendix C

### Materials

**Table C1.** English and Japanese Sentence Tokens Paired with Imagined Addressees.

English	Japanese	Addressee
When will you be in Ealing?	いついいリングにいる? [itsu iiringu-ni iru?]	Female formal
Where is the manual?	マヌあるわどこにある? [manuaru-wa doko-ni aru?]	Male formal
Why is he on the bed?	なんで彼わベッドの上にいる [nande kare-wa beddo-no ue-ni iru?]	Female informal
Why are we in a limousine?	なんでリイムジンの中にいる? [nande rimujin-no naka-ni iru?]	Male informal
You remembered Lil?	リルのことを思い出した？[ riru-no koto-wo omoida-shita?]	Female formal
You will lose Billy?	ビリーを失うことになる? [birii-wo ushinau koto ni naru?]	Male formal
You remembered Lillian?	ビルを失うことになる? [biru-wo ushinau koto ni naru?]	Female informal
You will lose Bil?	ビルを失うことになる? [biru-wo ushinau koto ni naru?]	Male informal
Did you say mellow or yellow?	メローとイエローのどっちをいったの？[meroo to ierro no dochi-wo itta no?]	Female formal
Is his name Miller or Mailer?	彼の生瀬わミラーと,マイラーのどっちなの？ [kare-no namae-wa miraa to mairaa no dochi nano?]	Male formal
Are you growing limes or lemons?	ライムとレモンのどっちーを育てるの？[raimu to remon no dochi-wo sodata-re no?]	Female informal
Did he say red or bed?	レッドとベッドのどっちを行ったの？ [reddo to beddo no dochi-wo itta no?]	Male informal
We remembered Lillian.	リリアンことを思い出し。 [ririan-no koto-wo omoidashita.]	Female formal
We remembered Lil.	リルのことを思い出した。 [riru-no koto-wo omoida-shita.]	Male formal
We will lose Billy.	ビルを失うことになる。 [biru-wo ushinau koto-ni naru.]	Female informal
We will lose Bil.	ビリーを失うことになる。 [birii wo ushinau koto ni naru.]	Male informal

## Appendix D

### Results (best-fit mixed-effects models and post hoc tables)

#### Cross-language differences

##### Pitch level

*Best-fit mixed-effects model* (significance level *p* < .05): mean F0 ~ Language + Sex of the participant + Testing Location + Language: Testing Location + (1| Participant)

**Table D1.** Post Hoc Pairwise Comparisons for the Significant Interaction Between Language and Location for the Mean F0 of the Two Languages of the Bilinguals.

Language	Testing location	Contrast	Estimate	*SE*	*t* ratio	*p* value
English		London—Tokyo	3.95	5.13	0.77	1
Japanese		London—Tokyo	–2.05	5.13	–0.4	1
	London	English—Japanese	14.6	1.04	13.97	<.0001
	Tokyo	English—Japanese	8.16	0.97	8.85	<.0001

*Note. p* values are Bonferroni-adjusted for multiple comparisons.

##### Pitch span

*Best-fit mixed-effects model* (significance level *p* < .05): 80% span ~ Language + Sex of the participant + Testing Location + Language: Sex of the participant + Language: Testing Location + Sex of the participant: Testing Location + Language: Sex of the participant: Testing Location + (1|Participant)

**Table D2.** Post Hoc Pairwise Comparisons for the Significant Interaction Between Language, Sex of the Participant, and Testing Location for the 80% Span of the Two Languages of the Bilinguals.

Testing location	Language	Sex of the participant	Contrast	Estimate	*SE*	*t* ratio	*p* value
London	English		Female–male	−0.02	0.70	−0.03	.977
Tokyo	English		Female–male	−1.76	0.75	−2.32	.289
London	Japanese		Female–male	−1.36	0.70	−1.94	.698
Tokyo	Japanese		Female–male	−1.97	0.75	−2.61	.142
	English	Female	London–Tokyo	0.91	0.64	1.42	.161
	Japanese	Female	London–Tokyo	0.90	0.64	1.41	.165
	English	Male	London–Tokyo	−0.83	0.90	−0.93	.358
	Japanese	Male	London–Tokyo	0.28	0.90	0.32	.754
London		Female	English–Japanese	−1.43	0.18	−7.89	<.0001
London		Male	English–Japanese	−2.76	0.24	−11.69	<.0001
Tokyo		Female	English–Japanese	−1.43	0.15	−9.45	<.0001
Tokyo		Male	English–Japanese	−1.65	0.28	−5.89	<.0001

*Note. p* values are Bonferroni-adjusted for multiple comparisons.

#### Expression of social meaning in English and Japanese

##### Pitch level

*English best-fit mixed-effects model* (significance level *p* < .025): mean F0 ~ Sex of the participant + Femininity scale (BSRI-short) + Masculinity scale (BSRI-short) + Sex of the participant: Femininity scale (BSRI-short) + Sex of the participant: Masculinity scale (BSRI-short) + (1|Participant)

**Table D3.** Post Hoc Analysis for the Significant Interaction Between Sex of the Participant and Scores on the Femininity Scale of the BSRI-Short for English Mean F0.

Sex of the participant	Femininity scale (BSRI-short)	Trend^a^	*SE*	*t* ratio	*p* value
Female		1.88	4.12	0.46	0.65
Male		−11.88	4.27	−2.78	.007

*Note*. *p* values are Bonferroni-adjusted for multiple comparisons.

a *Trend* is the estimate slope of the continuous variable obtained with the emmeans function emtrends (see, for example, https://rdrr.io/cran/emmeans/man/emtrends.html). In Table D3, the trend column reports the estimates of *slopes* of the continuous variable (Femininity scale of the BSRI-short) for each level of the factor Sex of the participant (female vs. male).

**Table:**

Contrast	Estimate	*SE*	*t* ratio	*p* value
Female–male	13.8	5.94	2.3	.024

**Table D4.** Post Hoc Analysis for the Significant Interaction Between Sex of the Participant and Scores on the Masculinity Scale of the BSRI-Short for English Mean F0.

Sex of the participant	Masculinity scale (BSRI-short)	Trend^a^	*SE*	*t* ratio	*p* value
Female		−10.87	3.97	−2.73	.008
Male		7.85	3.55	2.2	.032

*Note. p* values are Bonferroni-adjusted for multiple comparisons.

a In Table D4, the trend column reports the estimates of *slopes* of the continuous variable (Masculinity scale of the BSRI-short) for each level of the factor Sex of the participant (emale vs. male).

**Table:**

Contrast	Estimate	*SE*	*t* ratio	*p* value
Female–male	−18.7	5.33	−3.51	.001

*Japanese best-fit mixed-effects model* (significance level *p* < .025): mean F0 ~ Sex of the participant + Formality of the addressee + Sex of the addressee + Formality of the addressee: Sex of the addressee + (1|Participant)

**Table D5.** Post Hoc Pairwise Comparisons for the Significant Interaction Between Formality and Sex of the Addressee for Japanese Mean F0.

Formality of the addressee	Sex of the addressee	Contrast	Estimate	*SE*	*df*	*t* ratio	*p* value
Informal		Female–male	1.84	1.09	618	1.69	.365
Formal		Male–female	−5.44	1.09	618	−5.01	<.0001
	Female	Informal–formal	1.6	1.09	618	−1.47	.565
	Male	Informal–formal	−5.68	1.09	618	−5.22	<.0001

*Note. P* values are Bonferroni-adjusted for multiple comparisons.

## Notes

1. Throughout this paper, we used the terms pitch range and F0 range synonymously.

2. Note that neither Loveday (1981) nor Ohara (1992) specified the type of bilinguals they examined.

3. Images of the addressees cannot be included due to copyright reasons.

4. We carried out the feasibility test on two English monolinguals and two Japanese-English bilinguals. During the debriefing, the first author asked participants to report which of the images depicted formal versus informal people; they all indicated that people in business attire looked formal.

5. This phrasing is in line with recommendations from trans and non-binary activist work to avoid attributing a sex to people by the way they look (see Jas, 2020).

6. Note that maximal models only included the two individual gender identity scores specific to the language tested, that is, scores on the Bem Sex Role Inventory–short (BSRI-short) were entered for the analysis of English and scores on the Japanese Gender Role Index (JGRI) for the analysis of Japanese. This was done because (1) bilinguals have been reported to activate behavioral expression of personality appropriate to the corresponding linguistic-social context of the language they are speaking (Chen & Bond, 2010), and (2) we collected data in monolingual mode (see section 2.2).
